# COVID-19 Pandemic Impacts on Children with Developmental Disabilities: Service Disruption, Transition to Telehealth, and Child Wellbeing

**DOI:** 10.3390/ijerph19063259

**Published:** 2022-03-10

**Authors:** Saijun Zhang, Ying Hao, Yali Feng, Na Youn Lee

**Affiliations:** 1 Department of Social Work, University of Mississippi, Oxford, MS 38677, USA; szhang9@olemiss.edu (S.Z.); nlee2@olemiss.edu (N.Y.L.); 2 Department of Communication Sciences and Disorders, University of Mississippi, Oxford, MS 38677, USA; 3 Social Sciences, Health and Education Library, University of Illinois at Urbana-Champaign, Urbana, IL 61801, USA; yalifeng@illinois.edu

**Keywords:** children with developmental disabilities, service disruption, service transition, child wellbeing, COVID-19

## Abstract

The COVID-19 pandemic has resulted in substantial service disruption and transition from in-person services to telehealth for children with developmental disabilities. However, there is limited knowledge about the specific dimensions and consequences of the disruption and transition. This study aims to examine the extent of service disruption and transition, the experiences of client children and their caregivers with telehealth vis-à-vis in-person services, and the impacts of the disruption and transition on child wellbeing. The cross-sectional study collected data from parents of children with developmental disabilities using an online survey. McNemar’s tests were used to compare service changes before and after the pandemic outbreak, and multivariate analyses were used to examine how service changes were associated with child wellbeing. Results show that more than two-thirds of the children experienced reduction in service amount, and one-third lost services for more than two months in about five months into the pandemic. While telehealth had comparable features relative to in-person services, it had lower ratings with respect to diagnostic accuracy, treatment effectiveness, and rapport building. Service disruption/transition and social isolation were associated with behavioral and emotional deterioration in children. However, child and family stress may have confounded these adverse effects. We concluded that the magnitude of service disruption and transition was large in the first half year after the pandemic outbreak, and the amount and duration of service loss varied substantially across clients. Diagnostic accuracy, treatment efficacy, and rapport building were areas in which parents had major concerns toward telehealth relative to in-person services. However, such drawbacks may partially be due to the limited logistics in telehealth implementation during the pandemic. Service disruption and transition seemed to contribute to family stress, which played a direct role in eroding child wellbeing. Implications of these findings for future research and practices are discussed.

## 1. Introduction

The COVID-19 pandemic has severely disrupted the economy and people’s physical, psychological, and social wellbeing [1,2,3,4,5]. Children with developmental disabilities were particularly vulnerable during the pandemic [6] due to changes in routine services essential to facilitating development in these children [7,8]. Specifically, the pandemic’s impacts on services for children with developmental disabilities were mainly two-fold: service disruption, namely partial or full loss of services for at least a period of time, and service transition, from in-person services to telehealth to accommodate social distancing and other safety measures [9,10]. Extant studies have shown that service disruption and transition were prevalent among these children. However, their specific dimensions and the related consequences are less clear.

Developmental disabilities are prevalent and affect about 17% of children ages 3 to 17 years in the United States [11]. Developmental disabilities include attention deficit/hyperactivity disorder (ADHD), autism spectrum disorder (ASD), intellectual and physical disabilities, and other developmental delays [12]. Children with developmental disabilities often require routine services to facilitate their development, such as occupational therapy, physical therapy, mental health support, and speech-language services based in the community, school, and other settings [7,8]. Prior to the pandemic outbreak, these services were mostly delivered in person [10,13,14]. However, social distancing and other safety measures during the pandemic have seriously restricted provision of and access to such in-person services.

Service disruption and transition to telehealth for children with developmental disabilities was prevalent since the pandemic outbreak. Studies reported that one-third or more of children with developmental disabilities experienced at least partial service loss for a period of time [14,15,16,17]. Furthermore, about 60% to 80% of children who previously received in-person services transitioned to telehealth immediately or soon after the pandemic outbreak [10,14,17,18].

While extant research has described the prevalence of service disruption and transition in various settings, they often fall short of explicating the specifics of these changes. For example, how did the composition of in-person and telehealth service provision change from before the pandemic outbreak to after? Furthermore, service providers and client families had different responses to the sudden declaration of the pandemic and social distancing requirements due to varying resources and technical capacity [10]. However, there was limited attention to this variation (e.g., in the amount and duration) in service loss and transition since the pandemic outbreak. The knowledge is important for advancing our understanding of the pandemic’s impacts on service disruption and transition and subsequently on concerning children’s wellbeing.

Telehealth uses live video conferencing and other technologies to remotely deliver health care services [19]. Telehealth appears to show both strengths and weaknesses relative to traditional in-person services when serving children with developmental disabilities [20]. Studies comparing these two service models often highlight telehealth’s advantages in bridging geographical distance, increasing time flexibility, promoting cost-efficiency, and improving privacy by receiving services at home [20,21,22]. However, telehealth appeared to be inferior in communication efficiency and rapport building between clinicians and clients [22,23]. Its heavy reliance on technology may be particularly challenging for socioeconomically disadvantaged children and families who may have inadequacies in technology access and literacy [20].

Findings based on previous telehealth evaluation studies suggested that telehealth and in-person services are comparable in treatment efficacy [24,25,26]. These comparisons, however, were largely based on telehealth pilot programs and were restricted to small sample sizes. Given the large-scale transition from in-person services to telehealth during the pandemic, it is important to extend the understanding of differences between these two service models. However, research in this area is limited. Kowanda et al.’s (2021) study surveyed a sample of caregivers of children with neurodevelopmental genetic conditions during the pandemic and found that only about 30% to 60% of the caregivers considered telehealth to be meaningfully effective [17], but the study did not compare their telehealth experiences with their previous in-person service experiences. Another important aspect to consider is the sustainability of telehealth use. Because telehealth has been used as a substitute for in-person services extensively, it is important to examine how people’s experiences with telehealth may affect their views toward its future use. Previous research based on clinicians showed that clinicians had a high regard toward telehealth during the pandemic, but only a small proportion of them anticipated continuous telehealth use when the pandemic is over [27]. However, little is known about the clients’ perspectives.

The pandemic generates fear, frustration, stress, and other psychological adversities that can have a significant impact on child wellbeing [28]. This can be further exacerbated by extended isolation and loneliness due to social distancing and other safety measures [29]. The pandemic’s psychological impacts may be especially prominent among families of children with developmental disabilities. For example, Lee et al.’s (2021) study in the US found that more than one-third of the children experienced behavioral health degradation a few weeks into the pandemic, and that parents’ pervasive mental health problems, such as depression and anxiety, after the pandemic outbreak intensified children’s behavioral health problems [30]. Nonweiler et al.’s (2020) national study in the UK from April to June in 2020 showed that children with ADHD and ASD were three times more likely to have emotional and conduct problems than typically developing children [31]. Bentenuto et al. (2021) conducted a survey among parents of children with and without neurodevelopmental disorders in Italy during the lockdown period in February 2020 and found that parents of children with neurodevelopmental disorders reported a higher level of child problem behaviors [6]. Service disruption and transition may be a cause for the heightened behavioral and emotional deterioration [6,32]. While the pandemic’s damaging impacts on child wellbeing have been widely documented, there is a lack of knowledge on the magnitude of the degradation [6,31,32]. Furthermore, it is unclear how other influential factors such as family stress along with service disruption and transition influence this degradation.

Built upon extant research, the current study explored several dimensions of service disruption and transition after the COVID-19 pandemic outbreak among children with developmental disabilities. Specifically, the study examined: (a) To what extent services shifted from in-person format to telehealth format after the pandemic outbreak; (b) the amount and duration of service loss for the children due to the pandemic outbreak; (c) caregivers’ perceived differences between in-person services and telehealth services based on their experiences before and after the pandemic outbreak; and (d) the changes in child behavioral and emotional wellbeing after the pandemic outbreak, and how service disruption and transition as well as other factors were related to such changes.

## 2. Materials and Methods

### 2.1. Participants

The cross-sectional study used data from a sample of 101 parents/caregivers (caregivers hereafter) who had a child with developmental disabilities as defined by the CDC. From August to October 2020, we conducted an online survey using convenient sampling through the Mechanical Turk (MTurk), a popular research crowdsource platform based on Amazon’s online client population [33]. Using MTurk’s geographical setting, we targeted respondents who resided in the United States. To increase participation, we made four announcements with an interval of about two to three weeks on MTurk. The recruitment announcements stated the study purpose and that only adult caregivers of children with defined developmental disabilities would be eligible for the study, with the survey link being attached at the end. Starting from the second announcement, we applied a mechanism provided by the MTurk to limit the distributions to people who had not responded to the survey yet.

At the beginning of the survey, the respondents were presented with the informed consent form, and only those who consented could continue the survey. The respondents were then presented with two screening questions to determine their eligibility, with the first question asking whether they were 18 years or older and the second questions asking, “Are you the caregiver of a child (aged 0 to 17 years old) diagnosed with a developmental disability (e.g., ADHD, ASD, learning disability, intellectual disability, hearing loss, vision impairment, cerebral palsy and other developmental disabilities)?” Only respondents who responded “Yes” to both screening questions were allowed to continue to the following topic questions.

A total of 146 participants responded to the survey. We excluded one participant who responded “No” to the informed consent form, 12 who responded “No” to the screening questions, 26 who did not complete the survey (only answered a few questions at the beginning of the survey), and 6 who completed the survey in less than five minutes which caused concerns about response accuracy. Finally, a sample of 101 respondents with complete responses was used for the analyses. The study was reviewed and approved by the Institutional Review Board (IRB) at the first author’s university.

Among children reported by participating caregivers, the prevalence of ADHD, ASD, and other developmental disabilities was 57%, 28%, and 41%, respectively. Over half (51%) of the children were 0 to 8 years old, while the remaining were 9 to 12 (26%) and 13 to 17 (24%) years old. About three-quarters (73%) of the children were male and non-Hispanic White. Regarding parent and family characteristics, over half (56%) of the respondents were mothers, while 41% of the parents were 18 to 34 years old and the remaining were 35 to 54 years old. About two-thirds (68%) of the families had an annual income greater than $50,000, and about two-thirds (67%) of the parents were currently married.

### 2.2. Instruments

Data collection occurred a few months after the pandemic outbreak when empirical instruments for such data collection were scarce. To assess the changes, we developed a series of questions to catch relevant information by asking respondents the state of service use before and after the pandemic outbreak, or by asking the respondents to compare such changes. These instruments have not been empirically evaluated. However, their clear focuses and the good internal consistency (as shown by Cronbach alpha below) should provide confidence for the validity of the measurement.

One set of three questions was used to assess child service provision before the pandemic outbreak, led by “BEFORE the pandemic outbreak…” The first question asked, “Was the child enrolled in a special education program,” where the responses were *yes* (1) and *no* (0). The second question was “What was the child’s other treatment services for the disability,” where the responses were *in-person treatment with a service agency* (1), *telehealth treatment with a service agency* (2), and *no service* (3). The third question asked, “Did the child receive BOTH online and in-person services for the disability,” where the responses were *yes* (1) and *no* (0). Questions 2 and 3 were joined to create a variable *receiving other services before the pandemic outbreak*, which copied the three categories in question 2 but added a category of receiving both telehealth and in-person services to exclusively indicate any respondents who answered yes in question 3. One set of three corresponding questions that initiated with “AFTER the pandemic outbreak…” was used to assess child service provision after the pandemic break, and a variable *receiving other services after the pandemic outbreak* was similarly created.

Service amount change before and after the pandemic outbreak was measured with a question, “Comparing the services BEFORE and AFTER the pandemic outbreak (counting both in-person and telehealth services), you feel the amount of services for the child…”. The response options were, *has reduced greatly* (1), *has reduced moderately* (2), *has been similar* (3), *has increased moderately* (4), and *has increased greatly* (5). Response options 4 and 5 subsequently were combined to form a category *has increased moderately or greatly* because of the small proportion in option 5. Duration of service loss was measured with a question, “If the child was receiving treatment services for the disability before the pandemic outbreak, how long did the child lose services (counting both in-person and telehealth services)?” The options were *no service lost* (1), *losing service [for] less than 2 months* (2), *losing service [for] more than 2 months* (3), and *no service prior to the pandemic* (4).

The comparison between telehealth and in-person services by caregivers was measured in ten dimensions based on a leading question: “How would you rate the comparison between in-person services and telehealth for children with developmental disabilities.” Telehealth was compared to in-person services on ten dimensions including time flexibility, transportation convenience, diagnosis accuracy, treatment effect, communication clarity, ease of communication, rapport building with clinicians, comfortableness, privacy protection, and acceptance. Each dimension was rated on a five-point scale: *strongly disagree* (1), *somewhat disagree* (2), *neither agree nor disagree* (3), *somewhat agree* (4), and *strongly agree* (5), where greater values indicated more preference toward telehealth. A compound scale was derived from the mean of the ten items, with higher values indicating more favorable attitudes toward telehealth relative to in-person services. The Cronbach alpha for these items was 0.84. In addition, two summary questions were used to assess caregivers’ overall preference toward telehealth. One question asked, “Overall, comparing telehealth with in-person services, you would say telehealth is,” and the options were *much worse* (1), *worse* (2), *similar* (3), *better* (4), and *much better* (5). Another question asked, “How would you rate your likelihood of using telehealth in the future even if in-person services become normal,” and the response options were *very unlikely* (1), *unlikely* (2), *similar* (3), *more likely* (4), and *much more likely* (5).

Children’s behavioral and emotional change was measured using four items led by a question, “Comparing your child’s current status in a typical week with his/her status before the pandemic outbreak, how would you rate child behavior and emotion change below?” The items included “Becoming more aggressive or rebellious”, “Becoming more anxious or depressed”, “Becoming more restless and less concentrating”, and “Becoming more difficult to communicate”. The responses to these items were *not at all* (1), *a little* (2), *somewhat* (3), and *very much* (4). A compound scale was derived from the mean of the four items, with higher values indicating heightened behavioral and emotional problems. The Cronbach alpha for these items was 0.86. The compound scale was used as the dependent variable in the multivariate models when assessing factors associated with child behavioral and emotional wellbeing.

Potential causes for children’s behavioral and emotional change were measured using four items led by one question, “How would you rate the following causes due to the pandemic outbreak on child behavioral and emotional change?” The items included “Treatment services stopped or reduced”, “Services changed form in-person to online services”, “Isolation from friends, extended family members, and other social networks”, and “Child or family stress”. The responses to these items were *not at all* (1), *a little* (2), *somewhat* (3), and *very much* (4). Each of these four items was used as an independent variable in the OLS models to predict child behavioral and emotional wellbeing.

### 2.3. Data Analysis

We first used descriptive and bivariate analyses for univariate and bivariate analyses, respectively. We then compared service changes before and after the pandemic outbreak, where the McNemar’s tests were used to assess the differences based on binary outcomes to account for variability correlation. Finally, ordinary least squares (OLS) regression models were run to examine how children’s behavioral and emotional deterioration was associated with the concerning factors while controlling for covariates.

## 3. Results

Table 1 shows the changes in services for children with developmental disabilities before and after the pandemic outbreak. The results showed that there was a non-statistically significant change in children receiving services from special education programs before (64%) and after (60%) the pandemic outbreak. Other changes were all statistically significant at *p* < 0.001. The proportion of children receiving in-person services declined dramatically from 72% to 7%. Conversely, the percentage receiving telehealth and the combination of telehealth and in-person services increased from 3% and 6% to 43% and 18%, respectively. However, those who received no services increased from 19% to 33% (Table 1 top panel).

In terms of the service amount change and the duration of service loss due to the pandemic outbreak, about 1 in 6 (17%) of the children had not initiated services before the pandemic happened. Nearly one quarter (24%) had been receiving services and were able to continue the services. However, 60% of the children lost their services for a period, with more than one quarter (27%) losing services for a period of less than 2 months and one third (33%) losing services for more than 2 months (Table 1 bottom panel).

Table 2 shows caregivers’ perceptions of telehealth vis-à-vis in-person services. A value of 3 indicates caregivers viewing telehealth and in-person services as comparable on a specific dimension, while a value less than 3 indicates that telehealth was inferior and a value larger than 3 indicates that telehealth was superior. Caregivers on average viewed telehealth as superior in time flexibility and transportation convenience relative to in-person services. However, caregivers rated diagnostic accuracy, treatment effectiveness, rapport building with clinicians, and privacy protection (2.54 to 2.62) lower for telehealth relative to in-person services. Caregivers rated other items, including communication clarity, ease of communication, comfortableness, and acceptance (2.9 to 3.22), as similar between telehealth and in-person services. The composite scale based on the ten items had a mean of 2.87, which was similar to the overall rating of telehealth relative to in-person services (M = 2.51). Caregivers also indicated that they were between “unlikely” and “similarly” (M = 2.75) to use telehealth when in-person services are normal in the future.

Table 3 presents children’s behavioral and emotional changes after the pandemic outbreak and how caregivers identified the potential causes for these changes. Caregivers’ ratings of their children’s behavioral and emotional changes across the four dimensions ranged from 1.75 to 2.4, with the compound scale having a mean of 1.99. The ratings for the potential causes of children’s behavioral and emotional changes ranged from 2.22 to 2.89.

Table 4 presents the results from the OLS models predicting children’s behavioral and emotional deterioration, the dependent variable. Model 1 included only the control variables without the four potential causes (i.e., the independent variables). The r-squared value was 0.12, indicating the model explained 12% of the variation in children’s behavioral and emotional deterioration. The independent variables—service disruption and transition, social isolation, and child or family stress—were added to Models 2 to 4 in a stepwise manner. The r-squared values ranged from 0.28 to 0.56. The increases in the r-squared values suggested that these independent variables additionally explained the variation in the dependent variable. The full model (Model 4) showed that service disruption (*b* = 0.12, *p* = 0.028) and stress (*b* = 0.47, *p* < 0.001) were significantly associated with children’s behavioral and emotional deterioration. While service transition (*b* = 0.17, *p* = 0.003) was significant in Model 2 and social isolation (*b* = 0.31, *p* < 0.001) was significant in Model 3, they were not significantly associated with children’s behavioral and emotional deterioration in Model 4.

## 4. Discussion

This study investigates service disruption and transition to telehealth due to the pandemic for children with developmental disabilities based on caregivers’ reports. The study gives a historical snapshot of service delivery status during the pandemic. It also offers an opportunity to examine how service disruption and transition impacts child wellbeing and provides important implications for service development.

### 4.1. Service Disruption and Transition

The children reported by participating caregivers were generally able to continue services through special education programs, which had largely moved online during schools’ transformation to virtual learning after the pandemic outbreak [10]. In the current study, we paid more attention to the disruption and transition of services in the community since they were less known [10]. There was a large-scale transition from in-person services to telehealth, including the increase in the combined use of telehealth and in-person services. The transition was also accompanied by a substantial increase in children who were receiving no services (from about one-fifth to one-third). The findings were in line with those of previous studies based on various settings [10,14,16,17,27].

Our study also estimates the extent of service disruption in terms of amount and duration. About two-thirds of the children experienced service amount reduction, and about six months into the pandemic, one-third lost services for more than two months. This information extends our understanding of the pandemic’s significant influence on services for children with developmental disabilities. A small fraction (8%) of the caregivers reported service increases after the pandemic. This may be because some children started services after the pandemic and because the widespread implementation of telehealth helped some families overcome distance or other practical barriers that previously hindered service access. Future research can further explore these specific dimensions of service disruption and transition.

### 4.2. Telehealth and In-Person Comparison

The caregivers viewed transition from in-person services to telehealth as having both pros and cons. However, concerns around telehealth’s diagnostic accuracy, treatment effectiveness, and rapport building with clinicians generally reduced caregivers’ preferences for telehealth compared to in-person services. Caregivers viewed telehealth as having advantages in time flexibility and remote access, which aligned with the documentation in previous studies [20,21,22,27]. Caregivers viewed the two service methods as comparable in communication, comfortableness, and acceptance. The widespread adoption of videoconferencing and other telecommunication technologies during the pandemic may have greatly normalized telehealth and increased caregivers’ communication, comfortableness, and acceptance when using telehealth. However, it has been well noted that caregivers perceive telehealth to be lacking in rapport building [22,23]. Applying in-person services prior to telehealth, or adopting a combination of telehealth and in-person services may effectively facilitate rapport building between the clinicians and clients [22].

The findings further showed that caregivers perceived telehealth as less effective than in-person services in diagnosis and treatment. The findings corroborate prior knowledge that caregivers did not highly regard telehealth’s treatment effectiveness for children with developmental disabilities during the pandemic [17]. Conversely, previous telehealth evaluation studies based on pilot programs generally suggested comparable treatment efficacy for these two methods [24,25,26].

This evaluation discrepancy may be related to the contextual differences in telehealth practices. Although telehealth in the pilot programs typically were limited to small sample sizes, they usually engaged comparable participants in the studies to assess outcomes between telehealth and in-person service recipients. The large-scale transition from in-person services to telehealth due to the pandemic outbreak has made it possible to conduct a comparison of these two methods based on client families’ service experiences before and after the pandemic outbreak. During the abrupt transition, most clinicians and clients struggled with a lack of training and necessary resources [10]; also, telehealth may have been used indiscriminately to serve client children, regardless of its limitations with very young children, children with specific diagnoses, children in socioeconomically disadvantaged families, and children with other special situations [20,24,25]. While telehealth has served as a valuable alternative to in-person services during the pandemic, these limitations may have undermined their potential efficacy compared to in-person services. The current findings revealed caregivers’ perceptions of these two service methods in the specific context of the pandemic. However, future research should continue investigating the strengths and weaknesses of the two methods after the pandemic.

### 4.3. Deteriorations in Child Wellbeing and Related Factors

The findings showed that the concerning children had moderate emotional and behavioral deterioration several months into the pandemic outbreak. These findings aligned with the extensive documentation of deteriorations in child wellbeing during the pandemic in other studies [6,31,32].

Furthermore, the findings from the multivariate models suggested that service disruption and transition along with other pandemic-related factors were related to the deteriorations in children’s behaviors and emotions (dependent variable), which is consistent with previous findings that service disruption, social isolation, and child or family stress had a negative impact on the wellbeing of these children [34,35,36,37,38]. The final full model (Model 4 in Table 4) showed that only service disruption (service stop or reduction) and child or family stress were significantly associated with the dependent variable. That is, service transition from in-person services to telehealth and social isolation were not associated with the deteriorations in children’s behaviors and emotions. However, in the stepwise models (Model 2 and 3 in Table 4), both were significantly associated with the dependent variable and explained a sizable proportion of its variation. This suggested that stress may have a confounding effect with service transition and social isolation. Service transition and social isolation might contribute to child or family stress so that their effects on the dependent variable were largely absorbed by the stress variable, resulting in their effects not being shown in the full model when the stress variable was present. Future research examining factors related to child wellbeing may need to take this confounding effect into consideration. Individual and family adversities may contribute to stress in the family and subsequently affect child and family wellbeing. This may be especially true for families of children with developmental disabilities, given that the care of special-needs children can be exhausting and can seriously impair parental psychological wellbeing [39]. The findings also indicate the importance of integrating child disability treatment services with mental health support, including evidence-based online mental health support interventions [40], to address the accumulating stress among the concerning children and families to achieve the best outcomes.

The study’s limitations should be noted. First, the sample is not fully representative of caregivers of children with developmental disabilities given that it is predominantly white and perhaps biased toward higher-income households. Second, the small sample size restricted the sample’s representativeness and statistical power in the analyses. We were only able to recruit a moderate number of participants in the study, although MTurk may contain a large pool of respondents. This was likely because the targeted participants, caregivers of children with developmental disabilities, were a small subset of the pool. However, the small sample may suggest that participants abided by eligibility requirements, ensuring the reliability of the data. Third, the data were collected through the internet, which may have excluded the less connected and technologically savvy. It is thus likely that the realities of service disruption and transition, as well as their related consequences, were worse than what was portrayed in this study. Fourth, the current study examined caregivers’ perspectives toward telehealth and in-person services. The current data shows caregiver speculations and concerns. However, verifying these perspectives (e.g., concerns toward telehealth’s diagnostic accuracy, treatment effectiveness, and rapport building) is beyond the scope of the study and can be an important topic for future research. Finally, the examined relationships between service disruption/transition and children’s outcomes were associations based on cross-sectional data. Thus, we should be cautious in drawing causal conclusions about the relationships.

## 5. Conclusions

By surveying caregivers of children with developmental disabilities, the study estimated the extent of service disruption and transition from in-person services to telehealth due to the pandemic outbreak. More than two-thirds of the children experienced service amount reduction, and about one-third lost services for more than two months in about six months into the pandemic. Telehealth had advantages or comparable features in some aspects relative to in-person services, but caregivers gave it lower ratings on diagnostic accuracy, treatment effectiveness, and rapport building. The findings revealed the experiences of client children and their families with telehealth. Yet, the comparison of effectiveness should be viewed in the context of the pandemic as telehealth was abruptly implemented with various restrictions (e.g., the lack of training, resources, the need to accommodate all types of client children regardless of suitability). Caregivers reported moderate deterioration of child behavior and emotion due to the pandemic. Service disruption and transition as well as social isolation were associated with the deterioration. However, part of the effects, especially the effects in service transition and social isolation, may be confounded with those of child or family stress. The findings highlighted the importance of promptly resuming full services and optimizing service delivery for children with developmental disabilities, as well as developing service programs that integrate child disability services with mental health support to address stress in the families in order to achieve the best service outcomes.

## Figures and Tables

**Table 1:** Service changes for children with developmental disabilities before and after the pandemic outbreak (*n* = 101).

Variable	Pre Pandemic Outbreak (%)	Post Pandemic Outbreak (%)	McNemar’s Test ^a^	*p*-Value
Service Structure Transition				
Receiving special education services	*64*	*60*	*0.73*	*0.39*
*Receiving other services*				
In-person treatment services	72	7	66	<0.001
Telehealth treatment services	3	43	40	<0.001
Both telehealth and in-person services	6	18	7.2	0.007
No services	19	33	12.25	<0.001
Service Loss in Amount and Duration due to the Pandemic Outbreak				
*Service loss amount*	%			
Has reduced greatly	29			
Has reduced moderately	36			
Has been similar	28			
Has increased moderately	8			
*Service loss duration*				
No service lost	24			
Losing service less than 2 months	27			
Losing services more than 2 months	33			
No services prior to the pandemic	17			

Note. ^a^ Because the comparisons based on the pre- and post- pandemic outbreak were within the same group, McNemar’s tests were used to account for potential variability correlation.

**Table 2:** Caregiver perceptions of telehealth versus in-person services (*n* = 101).

Measure	Mean	Std	Min	Max
Compare with IN-PERSON services, TELE-HEALTH has more:				
1. Time flexibility	4.06	0.97	1	5
2. Transportation convenience	4.5	0.81	1	5
3. Diagnostic accuracy	2.54	0.82	1	5
4. Treatment effectiveness	2.59	0.91	1	5
5. Communication clarity	2.9	1.05	1	5
6. Ease of communication	3.18	1.14	1	5
7. Rapport building with clinicians	2.56	0.97	1	5
8. Comfortableness	3.17	1.12	1	5
9. Privacy protection	2.62	1.08	1	5
10. Acceptance	3.22	0.96	1	5
Telehealth preference scale (items 1 to 10; Cronbach alpha = 0.84)	2.87	0.63	1	5
11. Overall, comparing telehealth with in-person services, you would say telehealth is?	2.51	0.83	1	5
12. How would you rate your likelihood of using telehealth in the future even if in-person services become normal?	2.75	1.09	1	5

**Table 3:** Children’s behavioral and emotional changes after the pandemic outbreak and their potential causes (*n* = 101).

Measure	Mean	SD	Min	Max
Children’s behavioral and emotional changes after the pandemic outbreak:				
Becoming more aggressive or rebellious	1.75	0.86	1	4
Becoming more anxious or depressed	2.00	0.96	1	4
Becoming more restless and less concentrating	2.40	0.93	1	4
Becoming more difficult to communicate	1.80	0.89	1	4
Children’s behavioral and emotional scale	1.99	0.76	1	4
Potential Causes for children’s behavioral and emotional changes:				
Treatment services stopped or reduced	2.28	1.09	1	4
Service change from in-person to online services	2.49	1.23	1	4
Isolation from friends, extended family members, and other social networks	2.89	1.07	1	4
Child or family stress	2.22	0.93	1	4

**Table 4:** OLS regression models predicting children’s behavioral and emotional deterioration (*n* = 101).

Variable	Model 1			Model 2			Model 3			Model 4		
	*b*	SE	*p*	*b*	SE	*p*	*b*	SE	*p*	*b*	SE	*p*
Intercept	2.36	0.25	<0.001 ***	1.23	0.32	<0.001 ***	1.42	0.3	<0.001 ***	0.66	0.28	0.022 *
Age (0 to 8)												
9 to 12	0.2	0.19	0.298	0.34	0.17	0.048 *	0.1	0.17	0.558	0.37	0.14	0.011 *
13 to 17	−0.06	0.19	0.739	0.04	0.18	0.841	−0.1	0.18	0.552	0.04	0.14	0.791
Female	−0.11	0.17	0.535	−0.02	0.15	0.901	−0.16	0.15	0.315	−0.15	0.13	0.238
Non-Hispanic White	−0.41	0.17	0.02 *	−0.28	0.16	0.083	−0.23	0.16	0.158	−0.17	0.13	0.195
Disability												
ADHD	0.15	0.17	0.386	0.15	0.15	0.311	0.11	0.15	0.47	0.06	0.12	0.607
ASD	0.25	0.19	0.193	0.17	0.17	0.321	0.29	0.17	0.1	0.09	0.14	0.519
Family income > $50,000	−0.29	0.17	0.1	−0.16	0.16	0.317	−0.18	0.16	0.254	−0.14	0.13	0.256
Caregiver married	−0.07	0.17	0.688	−0.14	0.15	0.358	−0.22	0.16	0.172	−0.13	0.13	0.293
Potential Causes												
1. Service disruption				0.22	0.07	0.001 **				0.12	0.06	0.028 *
2. Service transition				0.17	0.06	0.003 **				0.06	0.05	0.203
3. Isolation							0.31	0.07	<0.001 ***	0.03	0.07	0.656
4. Stress										0.45	0.07	<0.001 ***
R-square	0.12			0.31			0.28			0.56		

* < 0.05, ** < 0.01, *** < 0.001.

## Data Availability

The study did not report any data.
